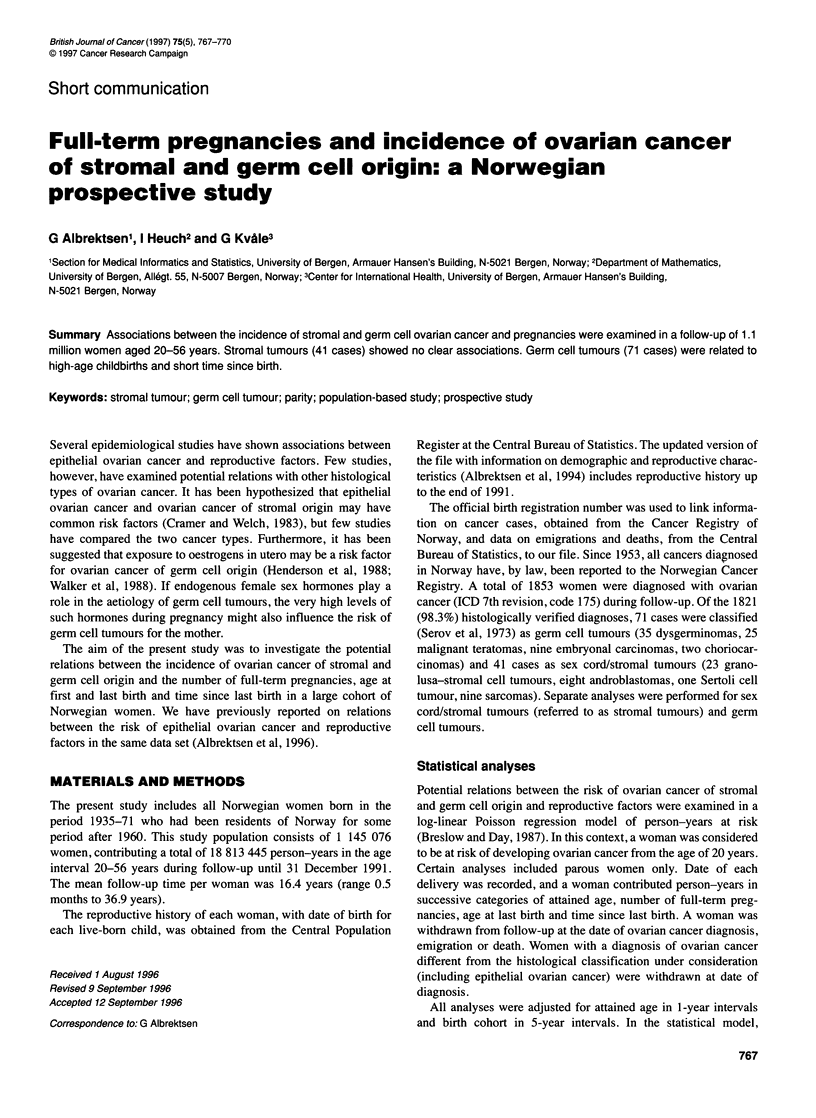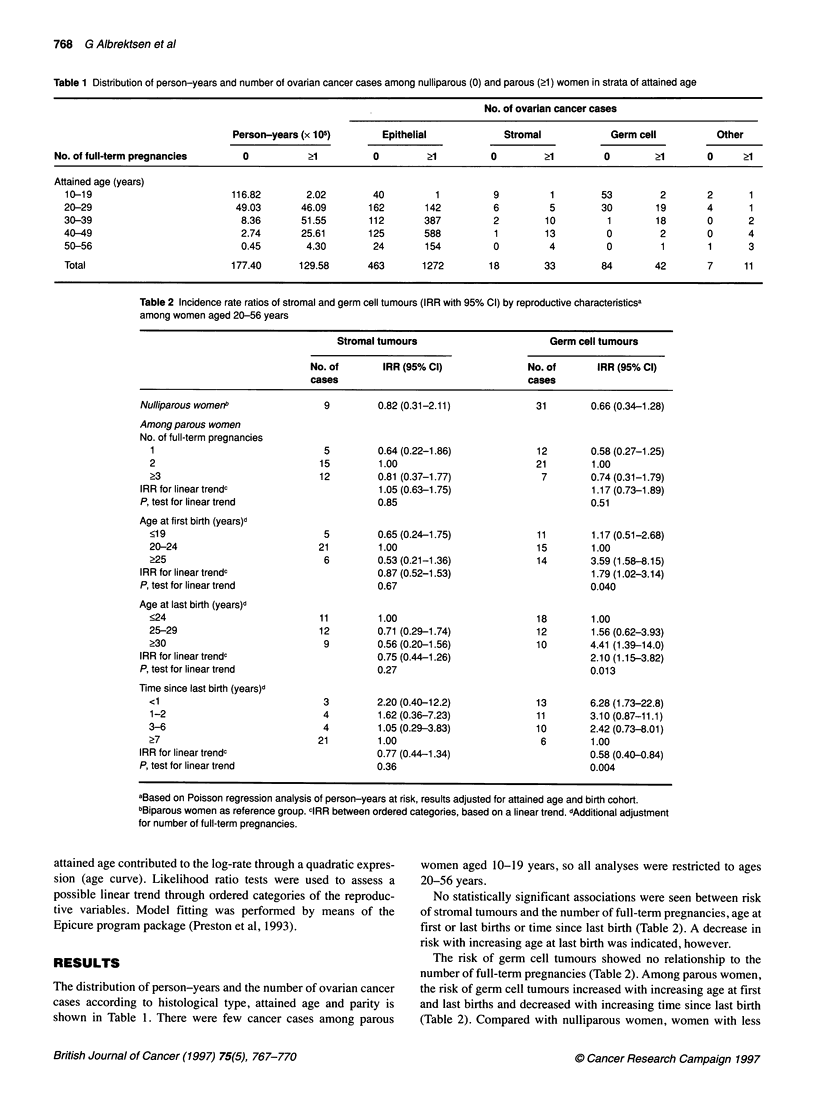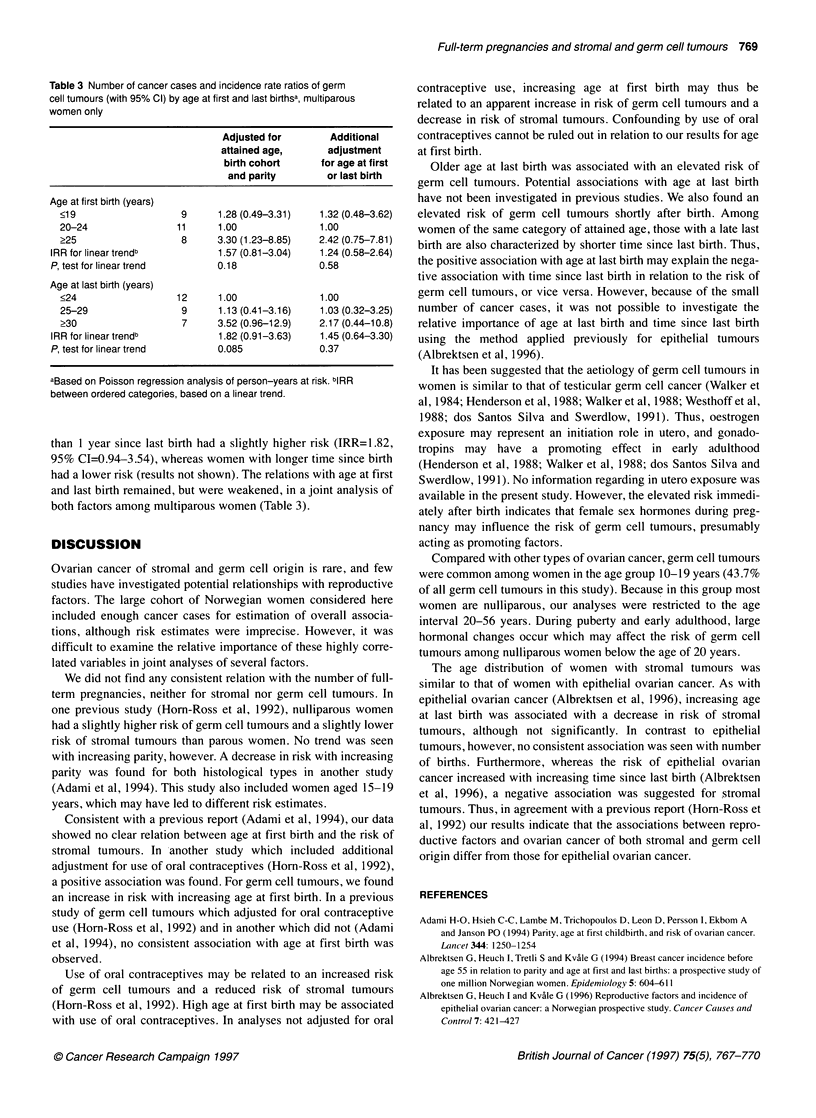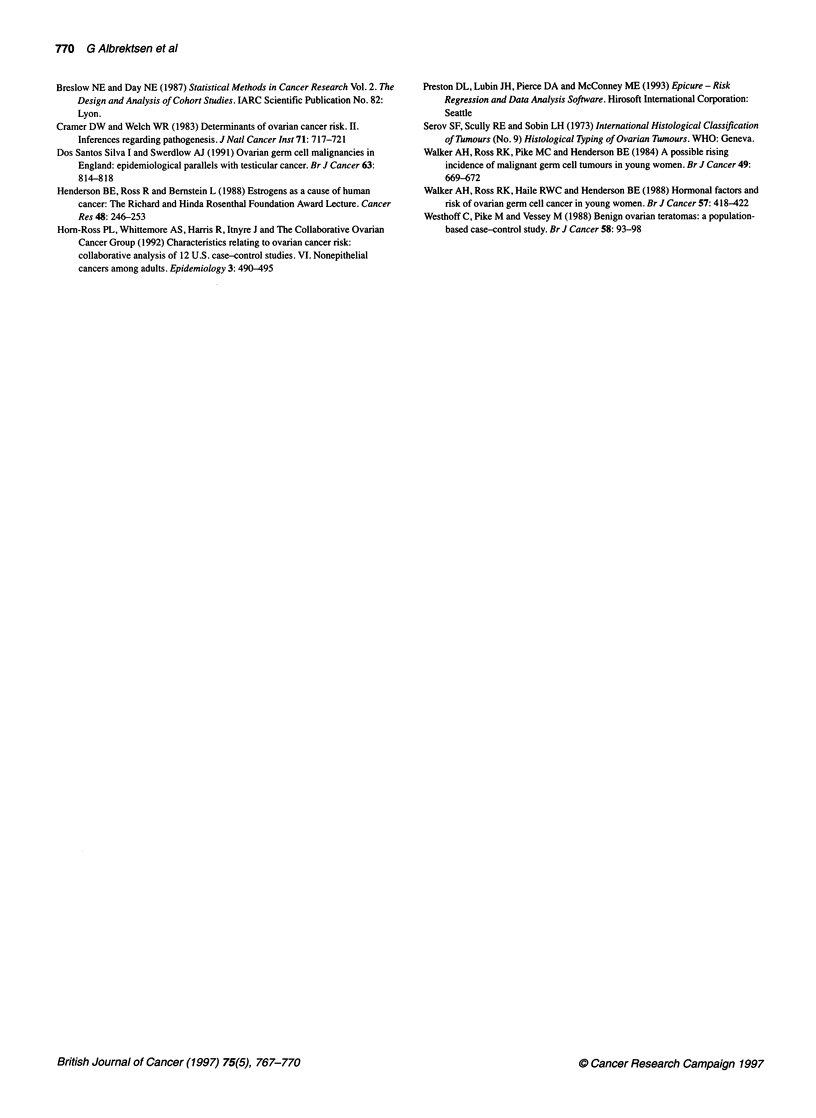# Full-term pregnancies and incidence of ovarian cancer of stromal and germ cell origin: a Norwegian prospective study.

**DOI:** 10.1038/bjc.1997.136

**Published:** 1997

**Authors:** G. Albrektsen, I. Heuch, G. Kvåle

**Affiliations:** Section for Medical Informatics and Statistics, University of Bergen, Norway.

## Abstract

Associations between the incidence of stromal and germ cell ovarian cancer and pregnancies were examined in a follow-up of 1.1 million women aged 20-56 years. Stromal tumours (41 cases) showed no clear associations. Germ cell tumours (71 cases) were related to high-age childbirths and short time since birth.


British Journal of Cancer (1997) 75(5), 767–770
© 1997 Cancer Research Campaign

Short communication

### Full-term pregnancies and incidence of ovarian cancer of stromal and germ cell origin: a Norwegian prospective study

G Albrektsen[1], I Heuch[2] and G Kvåle[3]

[1]Section for Medical Informatics and Statistics, University of Bergen, Armauer Hansen's Building, N-5021 Bergen, Norway; [2]Department of Mathematics, University of Bergen, Allégt. 55, N-5007 Bergen, Norway; [3]Center for International Health, University of Bergen, Armauer Hansen's Building, N-5021 Bergen, Norway

**Summary** Associations between the incidence of stromal and germ cell ovarian cancer and pregnancies were examined in a follow-up of 1.1 million women aged 20–56 years. Stromal tumours (41 cases) showed no clear associations. Germ cell tumours (71 cases) were related to high-age childbirths and short time since birth.

**Keywords:** stromal tumour; germ cell tumour; parity; population-based study; prospective study

Several epidemiological studies have shown associations between epithelial ovarian cancer and reproductive factors. Few studies, however, have examined potential relations with other histological types of ovarian cancer. It has been hypothesized that epithelial ovarian cancer and ovarian cancer of stromal origin may have common risk factors (Cramer and Welch, 1983), but few studies have compared the two cancer types. Furthermore, it has been suggested that exposure to oestrogens in utero may be a risk factor for ovarian cancer of germ cell origin (Henderson et al, 1988; Walker et al, 1988). If endogenous female sex hormones play a role in the aetiology of germ cell tumours, the very high levels of such hormones during pregnancy might also influence the risk of germ cell tumours for the mother.

The aim of the present study was to investigate the potential relations between the incidence of ovarian cancer of stromal and germ cell origin and the number of full-term pregnancies, age at first and last birth and time since last birth in a large cohort of Norwegian women. We have previously reported on relations between the risk of epithelial ovarian cancer and reproductive factors in the same data set (Albrektsen et al, 1996).

### MATERIALS AND METHODS

The present study includes all Norwegian women born in the period 1935–71 who had been residents of Norway for some period after 1960. This study population consists of 1 145 076 women, contributing a total of 18 813 445 person–years in the age interval 20–56 years during follow-up until 31 December 1991. The mean follow-up time per woman was 16.4 years (range 0.5 months to 36.9 years).

The reproductive history of each woman, with date of birth for each live-born child, was obtained from the Central Population Register at the Central Bureau of Statistics. The updated version of the file with information on demographic and reproductive characteristics (Albrektsen et al, 1994) includes reproductive history up to the end of 1991.

The official birth registration number was used to link information on cancer cases, obtained from the Cancer Registry of Norway, and data on emigrations and deaths, from the Central Bureau of Statistics, to our file. Since 1953, all cancers diagnosed in Norway have, by law, been reported to the Norwegian Cancer Registry. A total of 1853 women were diagnosed with ovarian cancer (ICD 7th revision, code 175) during follow-up. Of the 1821 (98.3%) histologically verified diagnoses, 71 cases were classified (Serov et al, 1973) as germ cell tumours (35 dysgerminomas, 25 malignant teratomas, nine embryonal carcinomas, two choriocarcinomas) and 41 cases as sex cord/stromal tumours (23 granulusa–stromal cell tumours, eight androblastomas, one Sertoli cell tumour, nine sarcomas). Separate analyses were performed for sex cord/stromal tumours (referred to as stromal tumours) and germ cell tumours.

### Statistical analyses

Potential relations between the risk of ovarian cancer of stromal and germ cell origin and reproductive factors were examined in a log-linear Poisson regression model of person–years at risk (Breslow and Day, 1987). In this context, a woman was considered to be at risk of developing ovarian cancer from the age of 20 years. Certain analyses included parous women only. Date of each delivery was recorded, and a woman contributed person–years in successive categories of attained age, number of full-term pregnancies, age at last birth and time since last birth. A woman was withdrawn from follow-up at the date of ovarian cancer diagnosis, emigration or death. Women with a diagnosis of ovarian cancer different from the histological classification under consideration (including epithelial ovarian cancer) were withdrawn at date of diagnosis.

All analyses were adjusted for attained age in 1-year intervals and birth cohort in 5-year intervals. In the statistical model,

*Received 1 August 1996*
*Revised 9 September 1996*
*Accepted 12 September 1996*

*Correspondence to:* G Albrektsen

**Table 1** Distribution of person–years and number of ovarian cancer cases among nulliparous (0) and parous (≥1) women in strata of attained age

| | Person–years (× 10⁵) | | No. of ovarian cancer cases | | | | | | | |
|---|---|---|---|---|---|---|---|---|---|---|
| | | | Epithelial | | Stromal | | Germ cell | | Other | |
| No. of full-term pregnancies | 0 | ≥1 | 0 | ≥1 | 0 | ≥1 | 0 | ≥1 | 0 | ≥1 |
| Attained age (years) | | | | | | | | | | |
| 10–19 | 116.82 | 2.02 | 40 | 1 | 9 | 1 | 53 | 2 | 2 | 1 |
| 20–29 | 49.03 | 46.09 | 162 | 142 | 6 | 5 | 30 | 19 | 4 | 1 |
| 30–39 | 8.36 | 51.55 | 112 | 387 | 2 | 10 | 1 | 18 | 0 | 2 |
| 40–49 | 2.74 | 25.61 | 125 | 588 | 1 | 13 | 0 | 2 | 0 | 4 |
| 50–56 | 0.45 | 4.30 | 24 | 154 | 0 | 4 | 0 | 1 | 1 | 3 |
| Total | 177.40 | 129.58 | 463 | 1272 | 18 | 33 | 84 | 42 | 7 | 11 |

**Table 2** Incidence rate ratios of stromal and germ cell tumours (IRR with 95% CI) by reproductive characteristics[a] among women aged 20–56 years

| | Stromal tumours | | Germ cell tumours | |
|---|---|---|---|---|
| | No. of cases | IRR (95% CI) | No. of cases | IRR (95% CI) |
| *Nulliparous women*[b] | 9 | 0.82 (0.31–2.11) | 31 | 0.66 (0.34–1.28) |
| *Among parous women* | | | | |
| No. of full-term pregnancies | | | | |
| 1 | 5 | 0.64 (0.22–1.86) | 12 | 0.58 (0.27–1.25) |
| 2 | 15 | 1.00 | 21 | 1.00 |
| ≥3 | 12 | 0.81 (0.37–1.77) | 7 | 0.74 (0.31–1.79) |
| IRR for linear trend[c] | | 1.05 (0.63–1.75) | | 1.17 (0.73–1.89) |
| *P*, test for linear trend | | 0.85 | | 0.51 |
| Age at first birth (years)[d] | | | | |
| ≤19 | 5 | 0.65 (0.24–1.75) | 11 | 1.17 (0.51–2.68) |
| 20–24 | 21 | 1.00 | 15 | 1.00 |
| ≥25 | 6 | 0.53 (0.21–1.36) | 14 | 3.59 (1.58–8.15) |
| IRR for linear trend[c] | | 0.87 (0.52–1.53) | | 1.79 (1.02–3.14) |
| *P*, test for linear trend | | 0.67 | | 0.040 |
| Age at last birth (years)[d] | | | | |
| ≤24 | 11 | 1.00 | 18 | 1.00 |
| 25–29 | 12 | 0.71 (0.29–1.74) | 12 | 1.56 (0.62–3.93) |
| ≥30 | 9 | 0.56 (0.20–1.56) | 10 | 4.41 (1.39–14.0) |
| IRR for linear trend[c] | | 0.75 (0.44–1.26) | | 2.10 (1.15–3.82) |
| *P*, test for linear trend | | 0.27 | | 0.013 |
| Time since last birth (years)[d] | | | | |
| <1 | 3 | 2.20 (0.40–12.2) | 13 | 6.28 (1.73–22.8) |
| 1–2 | 4 | 1.62 (0.36–7.23) | 11 | 3.10 (0.87–11.1) |
| 3–6 | 4 | 1.05 (0.29–3.83) | 10 | 2.42 (0.73–8.01) |
| ≥7 | 21 | 1.00 | 6 | 1.00 |
| IRR for linear trend[c] | | 0.77 (0.44–1.34) | | 0.58 (0.40–0.84) |
| *P*, test for linear trend | | 0.36 | | 0.004 |

[a]Based on Poisson regression analysis of person–years at risk, results adjusted for attained age and birth cohort. [b]Biparous women as reference group. [c]IRR between ordered categories, based on a linear trend. [d]Additional adjustment for number of full-term pregnancies.

attained age contributed to the log-rate through a quadratic expression (age curve). Likelihood ratio tests were used to assess a possible linear trend through ordered categories of the reproductive variables. Model fitting was performed by means of the Epicure program package (Preston et al, 1993).

### RESULTS

The distribution of person–years and the number of ovarian cancer cases according to histological type, attained age and parity is shown in Table 1. There were few cancer cases among parous women aged 10–19 years, so all analyses were restricted to ages 20–56 years.

No statistically significant associations were seen between risk of stromal tumours and the number of full-term pregnancies, age at first or last births or time since last birth (Table 2). A decrease in risk with increasing age at last birth was indicated, however.

The risk of germ cell tumours showed no relationship to the number of full-term pregnancies (Table 2). Among parous women, the risk of germ cell tumours increased with increasing age at first and last births and decreased with increasing time since last birth (Table 2). Compared with nulliparous women, women with less

© Cancer Research Campaign 1997

**Table 3** Number of cancer cases and incidence rate ratios of germ cell tumours (with 95% CI) by age at first and last births[a], multiparous women only

| | | Adjusted for attained age, birth cohort and parity | Additional adjustment for age at first or last birth |
|---|---|---|---|
| Age at first birth (years) | | | |
| ≤19 | 9 | 1.28 (0.49–3.31) | 1.32 (0.48–3.62) |
| 20–24 | 11 | 1.00 | 1.00 |
| ≥25 | 8 | 3.30 (1.23–8.85) | 2.42 (0.75–7.81) |
| IRR for linear trend[b] | | 1.57 (0.81–3.04) | 1.24 (0.58–2.64) |
| *P*, test for linear trend | | 0.18 | 0.58 |
| Age at last birth (years) | | | |
| ≤24 | 12 | 1.00 | 1.00 |
| 25–29 | 9 | 1.13 (0.41–3.16) | 1.03 (0.32–3.25) |
| ≥30 | 7 | 3.52 (0.96–12.9) | 2.17 (0.44–10.8) |
| IRR for linear trend[b] | | 1.82 (0.91–3.63) | 1.45 (0.64–3.30) |
| *P*, test for linear trend | | 0.085 | 0.37 |

[a]Based on Poisson regression analysis of person–years at risk. [b]IRR between ordered categories, based on a linear trend.

than 1 year since last birth had a slightly higher risk (IRR=1.82, 95% CI=0.94–3.54), whereas women with longer time since birth had a lower risk (results not shown). The relations with age at first and last birth remained, but were weakened, in a joint analysis of both factors among multiparous women (Table 3).

### DISCUSSION

Ovarian cancer of stromal and germ cell origin is rare, and few studies have investigated potential relationships with reproductive factors. The large cohort of Norwegian women considered here included enough cancer cases for estimation of overall associations, although risk estimates were imprecise. However, it was difficult to examine the relative importance of these highly correlated variables in joint analyses of several factors.

We did not find any consistent relation with the number of full-term pregnancies, neither for stromal nor germ cell tumours. In one previous study (Horn-Ross et al, 1992), nulliparous women had a slightly higher risk of germ cell tumours and a slightly lower risk of stromal tumours than parous women. No trend was seen with increasing parity, however. A decrease in risk with increasing parity was found for both histological types in another study (Adami et al, 1994). This study also included women aged 15–19 years, which may have led to different risk estimates.

Consistent with a previous report (Adami et al, 1994), our data showed no clear relation between age at first birth and the risk of stromal tumours. In another study which included additional adjustment for use of oral contraceptives (Horn-Ross et al, 1992), a positive association was found. For germ cell tumours, we found an increase in risk with increasing age at first birth. In a previous study of germ cell tumours which adjusted for oral contraceptive use (Horn-Ross et al, 1992) and in another which did not (Adami et al, 1994), no consistent association with age at first birth was observed.

Use of oral contraceptives may be related to an increased risk of germ cell tumours and a reduced risk of stromal tumours (Horn-Ross et al, 1992). High age at first birth may be associated with use of oral contraceptives. In analyses not adjusted for oral contraceptive use, increasing age at first birth may thus be related to an apparent increase in risk of germ cell tumours and a decrease in risk of stromal tumours. Confounding by use of oral contraceptives cannot be ruled out in relation to our results for age at first birth.

Older age at last birth was associated with an elevated risk of germ cell tumours. Potential associations with age at last birth have not been investigated in previous studies. We also found an elevated risk of germ cell tumours shortly after birth. Among women of the same category of attained age, those with a late last birth are also characterized by shorter time since last birth. Thus, the positive association with age at last birth may explain the negative association with time since last birth in relation to the risk of germ cell tumours, or vice versa. However, because of the small number of cancer cases, it was not possible to investigate the relative importance of age at last birth and time since last birth using the method applied previously for epithelial tumours (Albrektsen et al, 1996).

It has been suggested that the aetiology of germ cell tumours in women is similar to that of testicular germ cell cancer (Walker et al, 1984; Henderson et al, 1988; Walker et al, 1988; Westhoff et al, 1988; dos Santos Silva and Swerdlow, 1991). Thus, oestrogen exposure may represent an initiation role in utero, and gonadotropins may have a promoting effect in early adulthood (Henderson et al, 1988; Walker et al, 1988; dos Santos Silva and Swerdlow, 1991). No information regarding in utero exposure was available in the present study. However, the elevated risk immediately after birth indicates that female sex hormones during pregnancy may influence the risk of germ cell tumours, presumably acting as promoting factors.

Compared with other types of ovarian cancer, germ cell tumours were common among women in the age group 10–19 years (43.7% of all germ cell tumours in this study). Because in this group most women are nulliparous, our analyses were restricted to the age interval 20–56 years. During puberty and early adulthood, large hormonal changes occur which may affect the risk of germ cell tumours among nulliparous women below the age of 20 years.

The age distribution of women with stromal tumours was similar to that of women with epithelial ovarian cancer. As with epithelial ovarian cancer (Albrektsen et al, 1996), increasing age at last birth was associated with a decrease in risk of stromal tumours, although not significantly. In contrast to epithelial tumours, however, no consistent association was seen with number of births. Furthermore, whereas the risk of epithelial ovarian cancer increased with increasing time since last birth (Albrektsen et al, 1996), a negative association was suggested for stromal tumours. Thus, in agreement with a previous report (Horn-Ross et al, 1992) our results indicate that the associations between reproductive factors and ovarian cancer of both stromal and germ cell origin differ from those for epithelial ovarian cancer.

### REFERENCES

Adami H-O, Hsieh C-C, Lambe M, Trichopoulos D, Leon D, Persson I, Ekbom A and Janson PO (1994) Parity, age at first childbirth, and risk of ovarian cancer. *Lancet* **344**: 1250–1254

Albrektsen G, Heuch I, Tretli S and Kvåle G (1994) Breast cancer incidence before age 55 in relation to parity and age at first and last births: a prospective study of one million Norwegian women. *Epidemiology* **5**: 604–611

Albrektsen G, Heuch I and Kvåle G (1996) Reproductive factors and incidence of epithelial ovarian cancer: a Norwegian prospective study. *Cancer Causes and Control* **7**: 421–427

© Cancer Research Campaign 1997

Breslow NE and Day NE (1987) *Statistical Methods in Cancer Research* Vol. 2. *The Design and Analysis of Cohort Studies*. IARC Scientific Publication No. 82: Lyon.

Cramer DW and Welch WR (1983) Determinants of ovarian cancer risk. II. Inferences regarding pathogenesis. *J Natl Cancer Inst* **71**: 717–721

Dos Santos Silva I and Swerdlow AJ (1991) Ovarian germ cell malignancies in England: epidemiological parallels with testicular cancer. *Br J Cancer* **63**: 814–818

Henderson BE, Ross R and Bernstein L (1988) Estrogens as a cause of human cancer: The Richard and Hinda Rosenthal Foundation Award Lecture. *Cancer Res* **48**: 246–253

Horn-Ross PL, Whittemore AS, Harris R, Itnyre J and The Collaborative Ovarian Cancer Group (1992) Characteristics relating to ovarian cancer risk: collaborative analysis of 12 U.S. case–control studies. VI. Nonepithelial cancers among adults. *Epidemiology* **3**: 490–495

Preston DL, Lubin JH, Pierce DA and McConney ME (1993) *Epicure – Risk Regression and Data Analysis Software*. Hirosoft International Corporation: Seattle

Serov SF, Scully RE and Sobin LH (1973) *International Histological Classification of Tumours* (No. 9) *Histological Typing of Ovarian Tumours*. WHO: Geneva.

Walker AH, Ross RK, Pike MC and Henderson BE (1984) A possible rising incidence of malignant germ cell tumours in young women. *Br J Cancer* **49**: 669–672

Walker AH, Ross RK, Haile RWC and Henderson BE (1988) Hormonal factors and risk of ovarian germ cell cancer in young women. *Br J Cancer* **57**: 418–422

Westhoff C, Pike M and Vessey M (1988) Benign ovarian teratomas: a population-based case–control study. *Br J Cancer* **58**: 93–98

© Cancer Research Campaign 1997